# Economy in the Annual Report

**Published:** 1916-06-10

**Authors:** Thomas Ryan

**Affiliations:** Secretary. St. Mary's Hospital, Paddington, W.


*To the Editor of* THE HOSPITAL.

### Economy in the Annual Report.

*To the Editor of* THE HOSPITAL.

SIR,—With reference to your paragraph "Paper-saving at St. Mary's Hospital," under "Hospital and Institutional News," in your last issue, may I say that although this hospital followed Charing Cross Hospital in point of time in announcing in the papers the abbreviation of the annual report and the curtailment of its circulation, as a matter of war economy, it is erroneous to say that it followed the example of Charing Cross Hospital in instituting this economy. This was decided on in March, and communicated to the authorities of the King's Fund on April 5, without knowing what Charing Cross was doing or contemplating doing.

This is a matter of no consequence in itself, but the fact that two hospital boards, independently of each other, have cut down the size and circulation of their annual reports suggests that a substantial saving can be effected in this way, or the thing would not have been done.

I may add that I reckon St. Mary's has saved, in round figures, about £75—£45 in printing and £30 in postage—in addition to the economy of paper.

If Charing Cross Hospital will divulge how they managed to save between £200 and £300, St. Mary's will gladly, next year, "follow their example," if the war lasts as long.—I am, yours faithfully,

THOMAS RYAN, Secretary.

St. Mary's Hospital, Paddington, W., June 6, 1916.

